# The Physiopathological Role of the Exchangers Belonging to the SLC37 Family

**DOI:** 10.3389/fchem.2018.00122

**Published:** 2018-04-17

**Authors:** Anna Rita Cappello, Rosita Curcio, Rosamaria Lappano, Marcello Maggiolini, Vincenza Dolce

**Affiliations:** Department of Pharmacy, Health and Nutritional Sciences, University of Calabria, Rende, Italy

**Keywords:** SLC37A1-4, endoplasmic reticulum, glucose-6-phosphate translocase, G6PT deficiency, glycogen storage disease type Ib

## Abstract

The human *SLC37* gene family includes four proteins SLC37A1-4, localized in the endoplasmic reticulum (ER) membrane. They have been grouped into the SLC37 family due to their sequence homology to the bacterial organophosphate/phosphate (Pi) antiporter. SLC37A1-3 are the less characterized isoforms. SLC37A1 and SLC37A2 are Pi-linked glucose-6-phosphate (G6P) antiporters, catalyzing both homologous (Pi/Pi) and heterologous (G6P/Pi) exchanges, whereas SLC37A3 transport properties remain to be clarified. Furthermore, SLC37A1 is highly homologous to the bacterial glycerol 3-phosphate permeases, so it is supposed to transport also glycerol-3-phosphate. The physiological role of SLC37A1-3 is yet to be further investigated. SLC37A1 seems to be required for lipid biosynthesis in cancer cell lines, *SLC37A2* has been proposed as a vitamin D and a phospho-progesterone receptor target gene, while mutations in the *SLC37A3* gene appear to be associated with congenital hyperinsulinism of infancy. SLC37A4, also known as glucose-6-phosphate translocase (G6PT), transports G6P from the cytoplasm into the ER lumen, working in complex with either glucose-6-phosphatase-α (G6Pase-α) or G6Pase-β to hydrolyze intraluminal G6P to Pi and glucose. G6PT and G6Pase-β are ubiquitously expressed, whereas G6Pase-α is specifically expressed in the liver, kidney and intestine. G6PT/G6Pase-α complex activity regulates fasting blood glucose levels, whereas G6PT/G6Pase-β is required for neutrophil functions. G6PT deficiency is responsible for glycogen storage disease type Ib (GSD-Ib), an autosomal recessive disorder associated with both defective metabolic and myeloid phenotypes. Several kinds of mutations have been identified in the *SLC37A4* gene, affecting G6PT function. An increased autoimmunity risk for GSD-Ib patients has also been reported, moreover, SLC37A4 seems to be involved in autophagy.

## Introduction

The SLC37 family belongs to the largest human solute-carrier (SLC) superfamily, comprising more than 52 gene families, and over 400 membrane-bound proteins catalyzing the transport of metabolites across biological membranes (He et al., 2009; Perland and Fredriksson, 2017).

So far, four isoforms have been identified, named SLC37A1-4 (Bartoloni and Antonarakis, 2004; Chou and Mansfield, 2014). They are transmembrane proteins located in the endoplasmic reticulum (ER) membrane (Pan et al., 2011), and have been grouped into the SLC37 family due to their sequence homology to the bacterial organophosphate/phosphate (Pi) antiporter (Pao et al., 1998). Moreover, in the membrane transporter classification system included in the transport classification database, SLC37 carriers are reported to belong to the OPA family, classified as 2.A.1.4 (http://www.tcdb.org/). SLC37A1-4 translocases are also called sugar-phosphate exchangers SPX1-4 (Bartoloni et al., 2000; Takahashi et al., 2000; Bartoloni and Antonarakis, 2004), and are predicted to consist of 10–12 transmembrane domains (Chou and Mansfield, 2014).

SLC37A1, SLC37A2, and SLC37A4 are Pi-linked glucose-6-phosphate (G6P) antiporters, catalyzing both homologous (Pi/Pi) and heterologous (G6P/Pi) exchanges, and are inhibited to a very different extend by cholorogenic acid, while SLC37A3 transport activity is yet to be determinated (Chen et al., 2008; Pan et al., 2011).

SLC37A1, SLC37A2, and SLC37A3 are the less characterized SLC37 family members (Chou and Mansfield, 2014). SLC37A1 gene appears to be involved in breast (Iacopetta et al., 2010) and colorectal (Kikuchi et al., 2018) cancers. *SLC37A2* has been recently proposed as a vitamin D (Wilfinger et al., 2014; Saksa et al., 2015) and a phospho-progesterone receptor (Knutson et al., 2017) target gene. Moreover, in obese murine models its expression seems to be related to chronic inflammation that supports metabolic syndrome (Kim et al., 2007). In dairy cattle, a *SLC37A2* mutation appears to be responsible for increased female infertility due to embryonic death (Fritz et al., 2013; Reinartz and Dist, 2016). The *SLC37A3* gene has been possibly related to congenital hyperinsulinemia (Proverbio et al., 2013). Furthermore, this gene seems to be involved in epigenetic modifications, because its methylation level depends on fasting glucose blood levels, at least after a significant weight loss (Benton et al., 2015). SLC37A4, also known as glucose-6-phosphate translocase (G6PT), is the more extensively studied isoform, and is a member of the multicomponent glucose-6-phosphatase system (G6Pase-system). In the liver and kidney, the activity of this complex is required to maintain blood glucose homeostasis (Bartoloni and Antonarakis, 2004). Additionally, it supports neutrophil and macrophage functions (Chou and Mansfield, 2014).

In the past, G6Pase-system was believed to consist of a glucose-6-phosphatase, with its active site facing the ER lumen, and three translocases (known as T1-3). In detail, T1 mediated G6P import through the ER membrane, whereas T2 and T3 catalyzed Pi and glucose efflux from the ER cavity, respectively (Gerin et al., 2001). Moreover, the presence of a regulatory 21 kDa hepatic microsomal glucose-6-phosphatase stabilizing protein (SP) was also hypothesized (Burchell et al., 1985). The existence of T2, T3, and SP has never been proven.

Based on recent scientific literature, T1 corresponds to G6PT, and it works in complex with either glucose-6-phosphatase-α (G6Pase-α, also called G6PC1) or glucose-6-phosphatase-β (G6Pase-β, known as G6PC3; Chou et al., 2002).

G6Pase-α is specifically expressed in the liver, kidney, and intestine, and it hydrolyzes intraluminal G6P to Pi and glucose, then this sugar exits the cell and enters the bloodstream to maintain interprandial blood glucose homeostasis (Chou and Mansfield, 2014).

G6PT deficiency is responsible for glycogen storage disease type Ib (GSD-Ib, OMIM232220), whereas G6Pase-α impairment causes GSD type Ia (GSD-Ia, OMIM232200) (Chou et al., 2010a,b). Both disorders prevent the final steps of gluconeogenesis and glycogenolysis; as a result, endogenous glucose production is severely compromised creating metabolic impairment, consisting of fasting hypoglycemia, hyperlipidemia, hyperuricemia, lactic acidemia, growth retardation, and amassing of glycogen and fat in the liver and kidneys, causing hepatomegaly and nephromegaly, respectively (Chou et al., 2002, 2010b).

In neutrophils, G6PT is functionally coupled to the ubiquitous G6Pase-β, in order to support neutrophil and macrophage functions (Chou et al., 2010a,b; Jun et al., 2010). G6Pase-β deficiency results in severe congenital neutropenia (Boztug et al., 2009). This condition has been considered as a glycogen storage disease I related syndrome (GSD-Irs, OMIM 612541).

Unlike GSD-Ia, both GSD-Irs (Cheung et al., 2007; Jun et al., 2010; McDermott et al., 2010) and GSD-Ib (Kim et al., 2008; Jun et al., 2014) can cause neutropenia and myeloid dysfunction.

In this review, we focus on the physiopathological role of the SLC37A family members, in particular on the best characterized G6PT, highlighting its role in autophagy, an increased autoimmunity risk for GSD-Ib patients, as well as new promising therapeutic strategies for GSD-Ib.

## SLC37A1 family member

The human SLC37A1 protein, also knows as SPX1, is encoded by the *SLC37A1* gene (NM_018964), mapped to chromosome 21q22.3, and containing 19 coding exons and 7 untranslated exons. Alternative splicing origins different transcripts, although the predicted protein sequence is identical, consisting of 533 amino acids, with a calculated molecular weight of 58 kDa (Bartoloni et al., 2000). This latter contains a mitochondrial cleavage site, as well as both N- and C-terminal ER signals for the ER retention (Bartoloni et al., 2000). This protein displays 59, 35, and 22% sequence identity with the human SLC37A2, SLC37A3 and SLC37A4 proteins, respectively (Chou et al., 2013), and it is 86% identical to its mouse homolog (Bartoloni and Antonarakis, 2004). SLC37A1 and SLC37A2 isoforms are the most related, while all the remaining pairwise sequence comparisons between the other SLC37 family members show lower sequence identity; hence, it is feasible that they might have had an independent evolution.

The human SLC37A1 protein shares 30 and 71% sequence identity to bacterial GlpT and *Mus musculus* SLC37A2, respectively (Takahashi et al., 2000); suggesting that mammalian SLC37A1 could be able to transport glycerol-3-phosphate (G3P), probably catalyzing an heterologous G3P/Pi exchange; therefore its gene was also called G3PP (Bartoloni et al., 2000).

A G3P transport activity has never been demonstrated, althought SLC37A1 association with glycolipid metabolism has been suggested (Bartoloni and Antonarakis, 2004; Dolce et al., 2011). The hypothetical role of SLC37A1 as a G3P exchanger has been postulated in cancer cells (Iacopetta et al., 2010). In details, in estrogen receptor (ESR) negative SkBr3 breast cancer cells (Lappano et al., 2017), as well as in ESR positive endometrial Ishikawa tumor cells, the expression of the SLC37A1 transcript was proven to be upregulated by the epidermal growth factor (EGF), through the EGF receptor/mitogen-activated protein kinase/Fos transduction pathway. Notably, in the same work the SLC37A1 protein localization in the ER was also demonstrated, supporting the hypothesis that this protein could import G3P into the ER lumen to sustain phospholipid biosynthesis, required to promote cancer progression (Iacopetta et al., 2010).

The functional role of SLC37A1 was also investigated in other human diseases. Analyses led in patients affected by glycerol kinase deficiency-like syndrome, with glyceroluria but lacking mutations in the human glycerol kinase gene, found only non-pathogenetic sequence variants in the human *SLC37A1* gene, excluding its implication in this defect (Bartoloni et al., 2000).

Furthermore, this gene critically maps to autosomal recessive nonsyndromic deafness locus (DFNB10), on chromosome 21q22.3, but its involvement in the pathogenesis of this disease was also excluded by mutational analysis (Bartoloni et al., 2000).

More recently, *SLC37A1* upregulation (at the mRNA and protein levels) was found in patients with colorectal cancer (CRC), and it was associated with positive venous invasion, liver metastasis, and poor patient outcomes (Kikuchi et al., 2018). Moreover, in a colon cancer cell line, LS180, *SLC37A1* upregulation was positively correlated to Sialyl Lewis A and Sialyl Lewis X levels. These carbohydrate antigens are ligands for the adhesion molecule E selectin, and are responsible for the adhesion of cancer cells to the endothelium during metastasis, which is a typical process of cancer progression (Bartella et al., 2016; Iacopetta et al., 2017). Their positive modulation suggests that *SLC37A1* might play a key role in the hematogenous metastasis of CRC, even if the underlying mechanisms remain unclear (Kikuchi et al., 2018).

Currently, it is proven that SLC37A1 can catalyze both heterologous G6P/Pi and homologous Pi/Pi exchanges, but it is poorly sensitive to chlorogenic acid and is not functionally coupled to G6Pases. On this basis, it is unlikely that it is involved in blood glucose homeostasis (Pan et al., 2011), so its physiological role remains to be clarified.

SLC37A1 mRNA is ubiquitously expressed, although mainly in adult kidney, spleen, liver, small intestine, bone marrow, as well as in fetal liver, brain, and spleen (Bartoloni et al., 2000). In the main gluconeogenetic organs, liver, and kidney, the relative SLC37A1 transcript levels are rather low with respect to the SLC37A4 levels, since they represent <2%, whereas in the intestine and pancreas they constitute 60 and 69%, respectively, of those observed for SLC37A4 (Pan et al., 2011). In macrophages, the SLC37A1 transcript level is 43% of that found for SLC37A4, while in neutrophils, it is markedly (about 280 %) higher, suggesting that SLC37A1 might have a key role in such cells (Chou and Mansfield, 2014).

## SLC37A2 family member

*SLC37A2*, also knows as SPX2, was firstly identified in a work, conducted on mice and aimed to detect cAMP inducible genes playing a role in promoting cholesterol efflux from the macrophage cell line RAW264 via apoE and apoA1 (Takahashi et al., 2000). Two murine transcripts were identified, originated by the use of alternative polyadenylation sites. Both transcripts are highly expressed in bone marrow derived macrophages, and encode a 510 amino-acid protein with a predicted molecular weight of 55 kDa (Takahashi et al., 2000).

A further study showed that *SLC37A2* is abundantly expressed in murine macrophages, spleen and thymus, as well as in white adipose tissue (WAT) of genetically obese mouse models, since WAT is subject to considerable macrophage infiltrations, and this promotes obesity-associated chronic inflammation underlying metabolic syndrome and other comorbidities of obesity (Kim et al., 2007). The murine SLC37A2 protein undergoes post-translational modifications by N-linked glycosylation, and it migrates as a heterogeneous species of 50–75 kDa (Kim et al., 2007).

The human SLC37A2 protein is encoded by the *SLC37A2* gene (NM_198277), mapped to chromosome 11q24.2 and consisting of 18 coding exons. Alternative splicing originates four different transcripts. Only the longest isoform has been characterized (Pan et al., 2011). The corresponding human SLC37A2 transcript is expressed in murine liver, kidney, intestine, and pancreas, however the related expression levels are <4.5% of those found for SLC37A4 (Pan et al., 2011). Noticeably, the SLC37A2 transcript levels increase 46-fold during differentiation of human monocytic leukemia cells (THP-1) to macrophages (Kim et al., 2007).

The human SLC37A2 protein consists of 505 amino acids and displays 59, 36, and 23% sequence identity with the human SLC37A1, SLC37A3, and SLC37A4 proteins, respectively (Chou et al., 2013). Moreover, it is 90% identical to its mouse homolog (Bartoloni and Antonarakis, 2004). Like the murine protein, also the human protein is post-translationally modified by N-linked glycosylation.

The SLC37A2 protein is able to catalyze both G6P/Pi and Pi/Pi exchanges (Pan et al., 2011). Similarly to SLC37A1, the SLC37A2 transport activity is poorly sensitive to chlorogenic acid and the protein is not functionally coupled to G6Pases, as well as it seems not to be involved in blood glucose homeostasis (Pan et al., 2011). Hence, the functional role of SLC37A2 is yet to be understood. Recently, *SLC37A2* has been found as a vitamin D target gene (Wilfinger et al., 2014; Saksa et al., 2015). Vitamin D_3_ may affect gene regulation via the binding of its metabolite, 1α,25-dihydroxyvitamin D_3_ (1,25(OH)_2_D_3_), to the transcription factor vitamin D receptor (VDR). In monocytic and macrophage-like cells, the human *SLC37A2* gene contains a conserved VDR-binding site allowing such modulation, although only in monocytic cells *SLC37A2* is an early responding target gene, potentially useful as a biomarker of vitamin D_3_ status in the hematopoietic system (Wilfinger et al., 2014). In addition, in human peripheral blood mononuclear cells, changes in the expression of the *SLC37A2* gene, together with those of other primary vitamin D target genes, are systematically associated with the alteration in the circulating form of vitamin D_3_. Remarkably, during vitamin D_3_ supplementation in pre-diabetic subjects those features allow a distinction into high and low responder patients (Saksa et al., 2015).

Remarkably, in dairy cattle a deleterious homozygous mutation (g.28879810C>T) was detected in an aborted fetus. This mutation was predicted to introduce a premature stop codon, strongly impairing protein structure, and it was believed to be responsible for embryonic lethality (Reinartz and Dist, 2016). The same mutation, leading to embryonic lethal defects with increased female infertility was also detected in another study (Fritz et al., 2013).

Considering that the *SLC37A2* gene carries a VDR binding site, and that vitamin D_3_ may be involved in many biological pathways, such as calcium and phosphate homeostasis, cell growth, intracellular metabolism, as well as innate and adaptive immunity, embryonic death could depend on a deficit in such processes (Reinartz and Dist, 2016).

Recently, human *SLC37A2* has also been proposed as a phospho-Ser294 progesterone receptor (phospho-Ser294 PR) target gene (Knutson et al., 2017). PR Ser294 phosphorylation is a common event in breast cancer progression, and its activity is significantly associated with invasive lobular carcinoma. The runt-related transcription factor 2 (RUNX2) is an osteoblast differentiation transcription factor expressed in developing breast epithelial cells; it appears to be required in the regulation of phospho-Ser294 PR target genes. In this regard, human *SLC37A2* represents a good candidate as target gene, because it is expressed in monocytes, as well as in breast and cervical tissues, and it was found to contain multiple RUNX2 binding motifs immediately upstream and within the gene; moreover, its expression is proven to be upregulated by progestin in multiple cell line models (Knutson et al., 2017).

## SLC37A3 family member

The human SLC37A3 protein, also knows as SPX3, is the less characterized SLC37 family member. It is encoded by the *SLC37A3* gene (NM_207113), mapped to chromosome 7q34 and containing 17 coding exons. Alternative splicing originates three different transcripts (Bartoloni and Antonarakis, 2004).

One isoform, consisting of 494 amino acids, displays 35, 36, and 22% sequence identity with the human SLC37A1, SLC37A2, and SLC37A4 proteins, respectively (Chou et al., 2013), and it is 90% identical to its mouse and rat homologs (Bartoloni and Antonarakis, 2004). Even though SLC37A3 is an ER-associated protein, it fails to show an uptake activity (Pan et al., 2011), hence its functional properties remain to be clarified. Remarkably, the SLC37A3 transcript is extremely expressed in murine neutrophils, pancreas, and, to a lesser extent, in the liver, kidney, intestine, and macrophages (Pan et al., 2011; Chou et al., 2013), suggesting a possible functional role in the immune system and pancreas (Chou and Mansfield, 2014). In this latter regard, the human *SLC37A3* gene could contribute to the pathogenesis of congenital hyperinsulinism of infancy (CHI). In detail, a mutation in this gene was found in one patient with CHI in which the molecular basis of the disease remained unknown, highligting that it could be responsible for the dysregulation of insulin secretion (Proverbio et al., 2013), even if the biological role of SLC37A3 in pancreatic insulin secretion has never been clarified.

More recently, epigenetic mechanisms were demonstrated to modify the human *SLC37A3* gene, since a robust correlation between change in fasting glucose and DNA methylation level within the human *SLC37A3* gene was found in subcutaneous adipose, after gastric bypass followed by a significant weight loss (Benton et al., 2015). This could suggest a possible involvement of SLC37A3 in obesity-related metabolic dysfunction.

## SLC37A4 family member

SLC37A4 is the best functionally characterized SLC37 family member (Chen et al., 2000, 2002, 2008). The human protein is encoded by a single copy gene, *SLC37A4* (NM_001467, OMIM 602671), mapped to chromosome 11q23 (Annabi et al., 1998), containing nine coding exons (Marcolongo et al., 1998; Gerin et al., 1999; Hiraiwa et al., 1999), and firstly isolated from a human bladder tumor cDNA library (Gerin et al., 1997).

This protein displays 20, 25, and 26% sequence identity with bacterial protein UhpT, GlpT, and UhpC, respectively (Gerin et al., 1997). UhpT and GlpT are OPAs (Maloney and Wilson, 1996), while UhpC is a putative G6P receptor controlling UhpT expression (Island et al., 1992).

SLC37A4 protein is scarcely related to the other SLC37 family members, as it shares 22% amino acid sequence homology with both SLC37A1 and SLC37A3, and it is 23% homologous to SLC37A2 (Chou et al., 2013). The human SLC37A4 protein is highly conserved in other species. Murine and rat homologous proteins share 98% sequence homology, as well as 95 and 93% sequence homology to the human protein, respectively (Lin et al., 1998). Two human tissue-specific splicing isoforms have been identified, because alternative splicing of exon 7 leads to the expression of two transcripts, G6PT and variant G6PT (vG6PT), differing by the inclusion of a 66-bp exon 7 sequence in vG6PT, and encoding proteins of 429 and 451 amino acids, respectively (Gerin et al., 1997; Hiraiwa et al., 1999; Lin et al., 2000). Human vG6PT contains 22 additional amino acids, and it is active in microsomal G6P transport; it has been detected in the brain, heart and skeletal muscle (Lin et al., 2000). G6PT mRNA is ubiquitously expressed, although at the highest levels in the liver, kidney, intestine (Lin et al., 1998; Pan et al., 2011), and in haematopoietic progenitor cells (Ihara et al., 2000). The physiological implications of those different expression patterns remain unclear. In this regard, inclusion of exon 7 sequence might increase vG6PT sensitivity for degradation, since in mouse models the turnover rate of vG6PT seems to be increased during myogenesis of muscle cells (Shieh et al., 2007). Both G6PT and vG6PT appear to be similarly active in G6P transport (Lin et al., 2000), although the majority of studies used G6PT.

Human G6PT is a hydrophobic protein whose transmembrane topology has been long debated. Hydropathy profile analysis predicted either 10 (Hoffman and Stoffel, 1993) or 12 transmembrane domains (Gerin et al., 1997). Protease protection and glycosylation scanning assays suggested a 10-transmembrane domains model, with both N- and C-termini protruding on the cytoplasmic side of the ER membrane (Pan et al., 1999). Conversely, homology modeling proposed a model containing 12 transmembrane α-helices (Almqvist et al., 2004). More recently, glycosylation scanning and protease sensitivity studies have indicated that the 10-domains model is more probable (Pan et al., 2009).

G6PT biological function is to translocate G6P from the cytoplasm into the ER lumen, where it is hydrolyzed to glucose and Pi either by G6Pase-α (Lei et al., 1996; Chou et al., 2010a,b) or by G6Pase-β (Shieh et al., 2003; Chou et al., 2010a,b).

In the past, only one G6Pase isoform was known, expressed exclusively in the liver, kidney and intestine (Lin et al., 1998). In 2003, a second isoform, ubiquitously expressed, was discovered and called G6Pase-β (Shieh et al., 2003). Consequently, the original isoform was renamed G6Pase-α. Both proteins are transmembrane phosphohydrolases essential for the last step of gluconeogenesis. They have similar topology (Pan et al., 1998; Shieh et al., 2003) and mechanism of action as regards G6P hydrolysis (Ghosh et al., 2002, 2004); their active sites are located inside the ER lumen, hence both enzymes require to be coupled with a functional G6PT to hydrolyze intraluminal G6P. On the other hand, G6Pase activity is required in turn for an efficient G6P transport (Lei et al., 1996).

Since G6PT is ubiquitous, tissue expression profiles of G6Pase-α or G6Pase-β, and the resulting G6PT/G6Pase complexes, reflect the different GSD-Ia, -Ib or -Irs phenotypes.

On this basis, when the G6PT/G6Pase-α complex is present in the main gluconeogenic organs (liver, kidney, and intestine), G6Pase-α mutations cause a defective glucose production with impaired blood glucose homeostasis between meals, that is the first biochemical hallmark of GSD-Ia (Chou et al., 2010a,b).

In neutrophils and macrophages, the G6PT/G6Pase-β complex preserves energy homeostasis and functionality, hence G6Pase-β mutations are responsible for GSD-Irs, an autosomal recessive disorder characterized by neutropenia and neutrophil dysfunction (Chou et al., 2010a,b), often associated with congenital cardiac and uro-genital anomalies (Boztug et al., 2009).

On the other hand, G6PT mutations underlie GSD-Ib, which implies either impaired metabolism as in GSD-Ia, or neutropenia and neutrophil dysfunction as in GSD-Irs (Chou et al., 2010a,b), even if in neutrophils and macrophages G6PT expression levels are rather low (Chou et al., 2013).

## Physiopathological role of SLC37A4

SLC37A4, known as G6PT or SPX4, is able to catalize both homologous Pi/Pi and heterologous G6P/Pi exchanges between the ER lumen and the cytoplasm (Chen et al., 2008). Early studies led on intact liver microsomes showed that the high specificity of G6PT for G6P is responsible for substrate specificity of the G6PT/G6Pase-α complex, since G6Pase-α is less specific for G6P (Arion et al., 1972, 1975).

G6PT transport activity is specifically and strongly inhibited by chlorogenic acid (Arion et al., 1997, 1998; Hemmerle et al., 1997; Hiraiwa et al., 1999; Chen et al., 2008), acting as a reversible, competitive inhibitor. Also some chlorogenic acid derivatives, S3483 (Arion et al., 1998; Leuzzi et al., 2003), and S4048 (Herling et al., 1999), competitively inhibit G6PT, even more potently than chlorogenic acid. They have been used in studies concerning metabolic impairment in GSD-1 animal models (Bandsma et al., 2001; Grefhorst et al., 2010).

Several studies have established that G6P uptake activity needs not only an active G6PT, but also a functional G6Pase (Lei et al., 1996; Shieh et al., 2003; Chen et al., 2008; Pan et al., 2011). In this regard, hepatic microsomes isolated from G6Pase-α-deficient (GSD-Ia) mice, maintaining a functional G6PT, showed decreased G6P uptake activity, when compared to wild type hepatic microsomes (Lei et al., 1996). Accordingly, in a GSD-Ia mouse model, G6P transport activity could be restored by gene therapy supporting G6Pase-α function (Zingone et al., 2000). On this basis, functional coupling between G6PT and G6Pase-α became evident. A first explanation was suggested considering that G6PT provided the enzyme's substrate by importing cytoplasmic G6P into the ER lumen. Secondly, the physical interaction between G6Pase-α and G6PT, probably mediated by allosteric mechanisms, could support transport activity. This functional coupling was verified achieving functional cell-based activity assays for recombinant G6PT proteins, in order to measure G6P transport activity (Hiraiwa et al., 1999; Chen et al., 2000, 2002, 2008; Pan et al., 2011). According to these studies, it was demonstrated that microsomes expressing a functional G6Pase-α, but lacking an active G6PT (G6Pase-α+/+/G6PT –/–) showed little or no G6P uptake activity. In the same way, microsomes expressing an active G6PT but having a defective G6Pase-α (G6Pase-α –/– /G6PT+/+) exhibited poor G6P uptake rates, and microsomes expressing functional G6Pase-α and G6PT (G6Pase-α+/+/G6PT+/+) had strikingly increased G6P uptake rates (Chou and Mansfield, 2014). Furthermore, using a reconstitution procedure into proteoliposomes (Della Rocca et al., 2015; Curcio et al., 2016) preloaded with Pi, G6PT was proven to be an antiporter able to efficiently exchange G6P/Pi, without needing for a G6Pase-α coexpression (Chen et al., 2008). Those evidences suggested that G6Pase-α coexpression might increase intraluminal Pi concentration, in order to create a driving Pi gradient, useful for supporting G6PT antiporter activity. Cell-based assays and functional reconstitution into proteoliposomes were also successfully employed to characterize 23 SLC37A4 mutations identified in GSD-Ib patients (Chen et al., 2008).

## SLC37A4 and autophagy

Recently, SLC37A4 was identified as a key activator of autophagy pathway, able to negatively regulate mammalian target of rapamycin complex 1 (mTORC1) activity (Ahn et al., 2015). Autophagy is activated under nutrient deficiency to preserve cell homeostasis, and its deficit is associated with several human diseases (Jiang and Mizushima, 2014). Amino acids deprivation induces autophagy by inhibiting mTORC1 (Sancak et al., 2010), conversely, mTORC1 inhibits autophagy by phosphorylating unc-51 like autophagy activating kinase 1 (ULK1) (Jung et al., 2009) and autophagy/beclin-1 regulator 1, in order to ubiquitinate ULK1 for degradation (Nazio et al., 2013). Autophagy can also be triggered by ER stresses (Kouroku et al., 2007) or hypoxia (Bellot et al., 2009). On the other hand, prolonged ER stress leads to the inhibition of autophagy flux (Lee H. et al., 2012). Among the several autophagy-related genes (ATGs), ULK1 plays a key role, since it encodes a serine/threonine kinase essential for the initiation step of autophagy, because it forms complexes that are mainly controlled by mTORC 1 (Ganley et al., 2009) and 5′ AMP-activated protein kinase (AMPK) (Kim et al., 2011).

Among the ATGs encoded proteins, ATG9 is a membrane polypeptide depending on ULK1 activity, that regulates autophagosomes biogenesis by delivering them membrane source derived from the *trans* Golgi network (Young et al., 2006). SLC37A4 seems to promote the initiation step of autophagy acting upstream of mTORC1. In detail, SLC37A4 overexpression increases the interaction between N-terminal Venus-tagged ULK1 (ULK1-VN) and C-terminal Venus-tagged ATG9 (ATG9-VC), improving autophagic flux independent of G6PT transport activity (Ahn et al., 2015).

Previous studies demonstrated that G6PT-mTORC1 signaling is essential in promoting autophagy in hepatic cell lines, in addition, mTORC1 failure is often related to metabolic diseases, including type 2 diabetes and cancer (Zoncu et al., 2011). In this regard, it was proposed that this translocase could affect mTORC1 function through calcium mobilization (Chen et al., 2003). Furthermore, it was also suggested that G6PT could modulate mTORC1 through AMPK, which in turn is an energy sensor activated in response to augmented cellular AMP, ADP or calcium levels (Mihaylova and Shaw, 2011). Since AMPK can regulate autophagy through either direct ULK1 phosphorylation or mTORC1 inhibition (Hawley et al., 2005), disruption of calcium mobilization due to SLC37A4 dysfunction might influence both AMPK and mTORC1, leading to autophagy inhibition (Ahn et al., 2015).

## SLC37A4 defect leads to GSD-Ib

SLC37A4 is the G6PT shared by the G6PT/G6Pase-α or -β complexes and responsible for GSD-Ib (Chou et al., 2002, 2010b; Chou and Mansfield, 2014).

Early studies based on the activity of the G6PT/G6Pase-α complex suggested the existence of five GSD-I subtypes, referred to as Ia (affecting the G6Pase catalytic subunit), Ib (affecting G6PT), IaSP, Ic, and Id, believed to arise from T2, T3, and SP deficiency, respectively (Lei et al., 1995; Matern et al., 2002). Furthermore, G6Pase-β deficit was responsible for the onset of GSD-Irs (Boztug et al., 2009).

In the past, partial kinetic analysis demonstrated a deficit of Pi export from the microsomal lumen, suggesting the existence of a third form of GSD-I, called GSD-Ic (OMIM 232240), caused by the involvement of a third gene postulated in the pathogenesis of the disease (Nordlie et al., 1983). Subsequently, genotyping studies found out detrimental mutations in the human SLC37A4 gene (Veiga-da-Cunha et al., 1998; Galli et al., 1999; Janecke et al., 2000), therefore it was confirmed that either GSD-Ib or -Ic were caused by mutations occurring in the same gene (Veiga-da-Cunha et al., 1999). Additional defects, reported in patients, affected either microsomal glucose translocation (Lei et al., 1995), or SP, a hypothetical 21-kD protein, able to stabilize the G6Pase catalytic unit *in vitro* (Burchell et al., 1985). These conditions were initially classified as GSD-Id and GSD-IaSP (Burchell and Waddell, 1990), respectively. A patient diagnosed with GSD-IaSP was found to be homozygous for a *G6Pase* mutation, so GSD-IaSP was reclassified as GSD-Ia (Lei et al., 1995). In the same way, the diagnosis of GSD-Id was withdrawn, because this disorder was caused by a single mutation found in the human *SLC37A4* gene (Veiga-da-Cunha et al., 1999, 2000). As a result, GSD-Ib was implicated in all the reported cases of non-GSD-Ia (Chou et al., 2010b).

G6PT deficiency causes GSD-Ib, an autosomal recessively inherited disease, involving ~20% of all GSD-I patients (Chou et al., 2002, 2010b). This disorder is not limited to any racial or ethnic group, although the prevalence of some mutations is higher in whites and Japanese ethnicity (Chou et al., 2010b).

**Figure 1:**
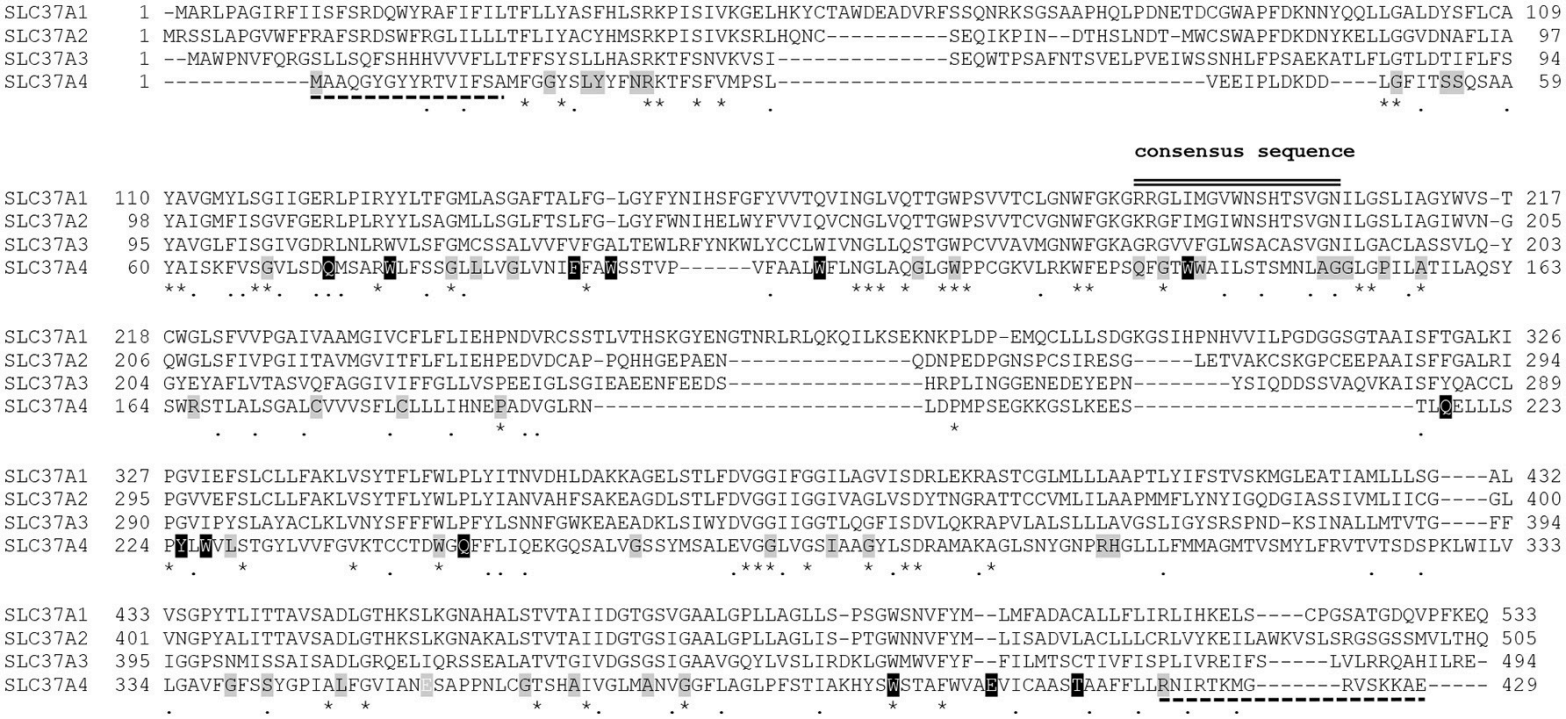
Alignment of the amino acid sequences of human SLC37A1, SLC37A2, SLC37A3, and SLC37A4 showing the location of nonsense and missense mutations identified in GSD-Ib patients. The aligned amino acid sequences are GENBANK accession numbers NP_061837.3 (SLC37A1), NP_938018.1 (SLC37A2), AAH46567.1 (SLC37A3), and CAG33014.1 (SLC37A4). Sequence conservation is indicated by an asterisk for identical residues, a dot for conserved substitutions, and a gap for non-conserved residues. The organo-phosphate/Pi antiporter family consensus sequence, ProSite PDOC00726, shared by the SLC37 family members is indicated by black double lines. Dashed black lines show lacking residues at the N- or C- terminal end in mutants MIV and R415X, respectively. Nonsense and missense mutations are highlighted in black or gray, respectively. Alignment has been performed by ClustalW.

**Figure 2:**
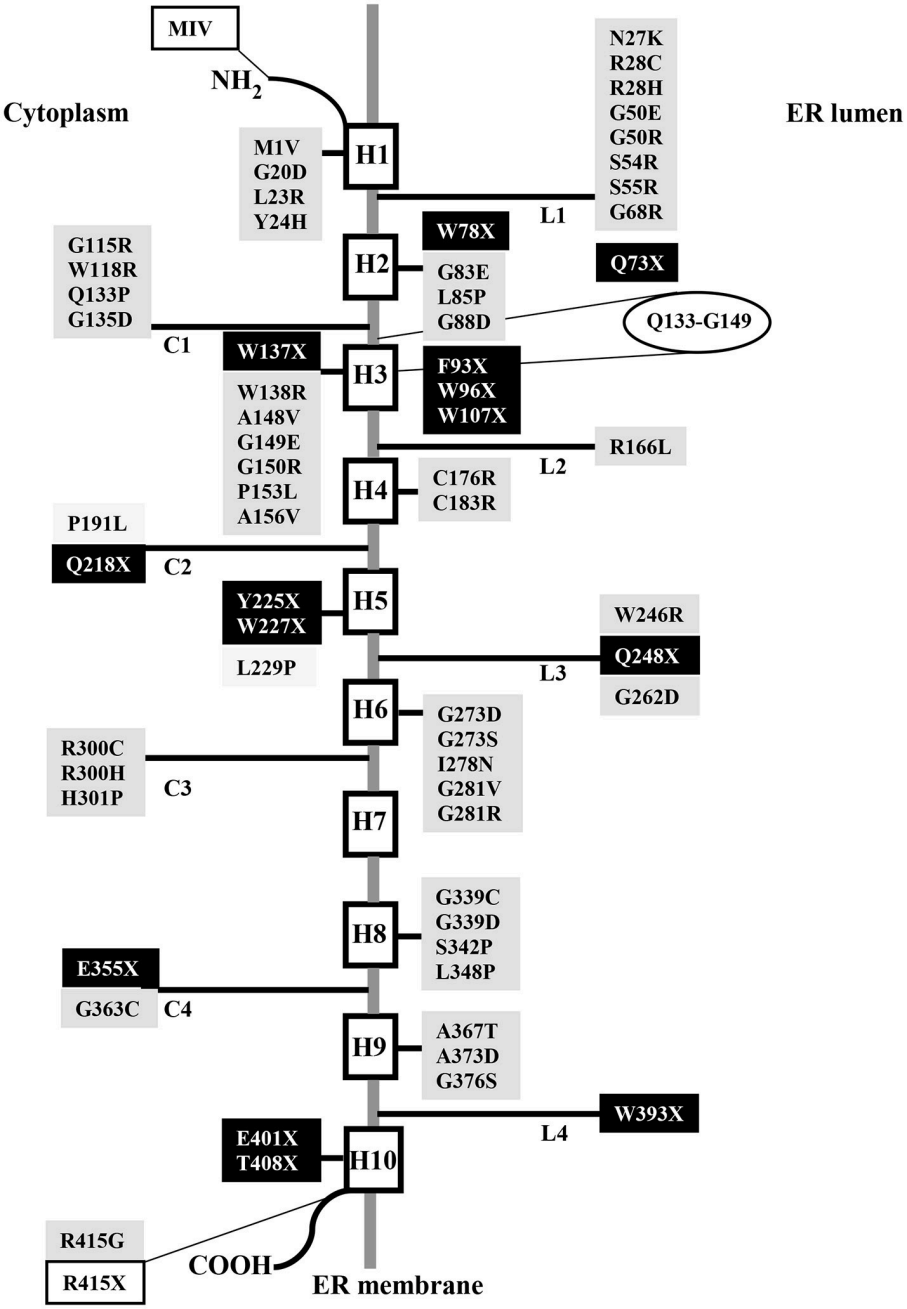
Schematic topological model of human G6PT displaying nonsense and missense mutations identified in GSD-Ib patients. Nonsense and missense mutations are highlighted in black or gray, respectively. The extension of the consensus sequence is reported in an ellipse. White boxes represent mutations that eliminate the N- or C- terminal domain.

Up to date, 110 separate mutations have been identified in the *SLC37A4* gene of GSD-Ib and non-GSD-Ia studied patients, including 61 missense/nonsense 1 regulatory and 17 splicing mutations, 29 small insertion/deletions, and 2 gross deletions (http://www.hgmd.cf.ac.uk/ac/gene.php?gene=SLC37A4). They were distributed throughout the gene. The missense mutation c.443C>T (A148V) seems to be restricted to Korean population, since it has not been reported in other ethnic groups (Rihwa et al., 2017). Several residues critical for G6PT function reside in the consensus sequence shared by the SLC37 family members (Figure 1), as well as by other OPA family members (Chou and Mansfield, 2014). In the topological model proposed for G6PT according to glycosylation scanning and protease sensitivity studies (Pan et al., 2009), many mutations are located in the first ER luminal loop (Figure 2). Here, a conserved arginine (R28), corresponding to R46 in UhpT and essential for activity (Lloyd and Kadner, 1990), is believed to constitute part of the substrate binding site and is required for G6P transport (Pan et al., 2009). Furthermore, N-terminal residues and helix 1 play a key role in transport activity, because the N-terminal mutation called MIV, lacking the N-terminal domain (residues1-7) and the first part of helix 1 (residues 8-16), abolishes transport function (Chen et al., 2002), but it does not interfere with G6PT stability (Chou and Mansfield, 2014). Conversely, the C-terminal domain deeply affects protein stability, since the nonsense mutation R415X, eliminating the whole cytoplasmic tail, causes a more rapid G6PT degradation with respect to the wild-type (Chen et al., 2000). Moreover, integrity of helix 10 is structurally important, because nonsense mutations E401X and T408X reduce G6PT expression and affect its folding (Chou and Mansfield, 2014).

## SLC37A4 defect: biochemical features and clinical phenotypes

**Figure 3:**
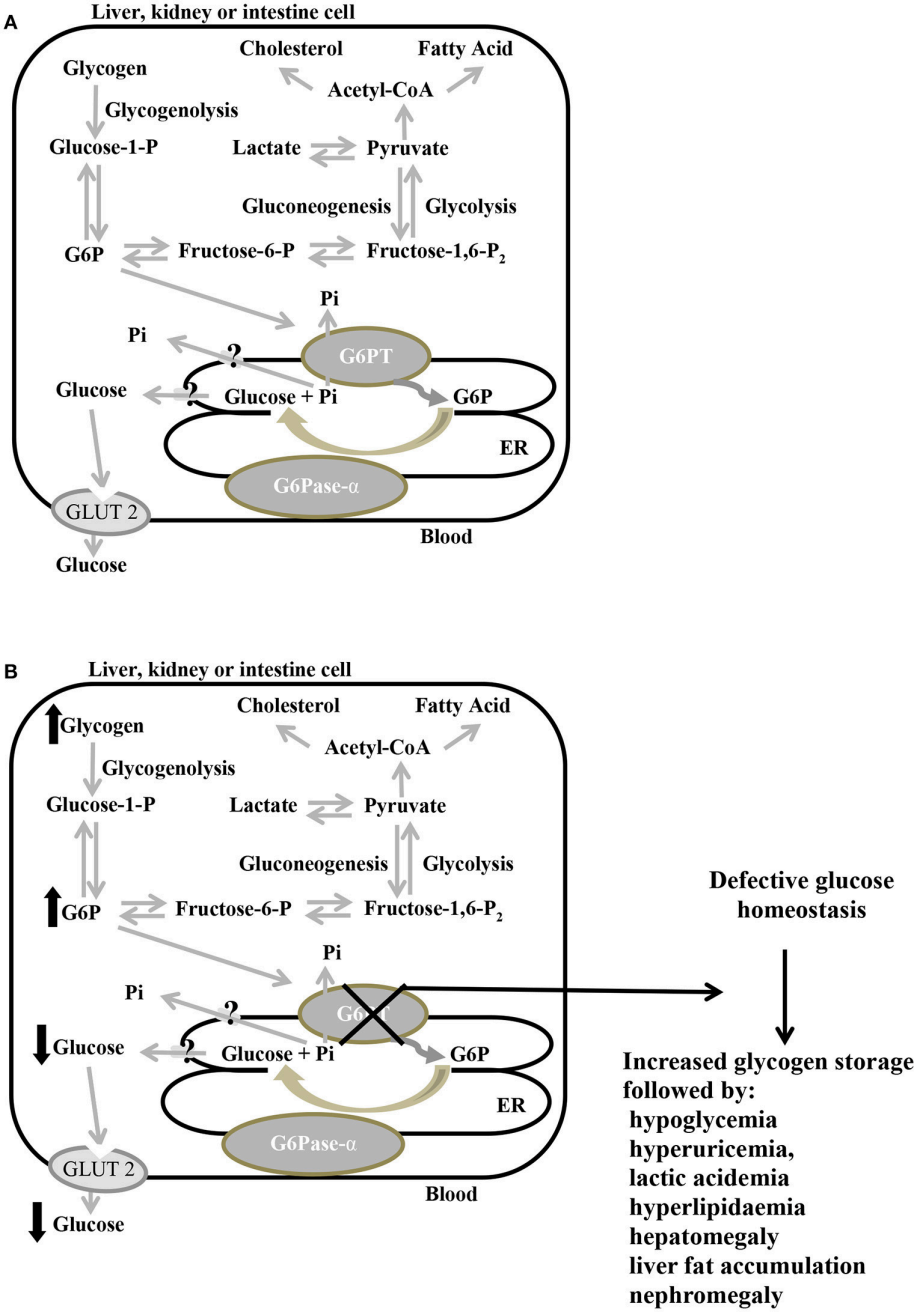
Primary metabolic pathways of G6P in the liver, kidney, and intestine, in normal **(A)** and defective G6PT **(B)** cells. Schematic cell harboring an extended endoplasmic reticulum (ER). G6Pase-α and G6PT are embedded in the ER membrane; the glucose transporter GLUT2 is embedded in the plasma membrane. Black arrows indicate metabolic changes due to defective SLC37A4. G6P, glucose-6-phosphate; G6Pase-α, glucose-6-phosphatase-α; G6PT, glucose-6-phosphate translocase; GLUT2, glucose transporter 2; P, phosphate; Pi, inorganic phosphate.

GSD-Ib-related symptoms are associated with both defective metabolic and myeloid phenotypes (Chou et al., 2010a,b). GSD-Ib metabolic phenotype is shared with GSD-Ia. Interprandial blood glucose homeostasis is controlled by the liver, the principal gluconeogenic organ, and to a lesser extent, by the kidney and intestine. Between meals, G6P produced in these organs during gluconeogenesis and glycogenolysis is imported into the ER lumen by G6PT, where it is hydrolyzed by G6Pase-α to produce glucose, then exported back into the bloodstream (Figure 3A; Chen, 2001; Chou et al., 2002). Since G6PT, as well as G6Pase-α, are abundantly expressed in gluconeogenic organs, when G6PT is defective the G6PT/G6Pase-α complex activity is defective. As a result of an inadequate glucose production, patients suffer from fasting hypoglycemia. At the same time, G6P cytoplasmic elevation leads to an abnormal storage of glycogen, which causes progressive nephromegaly and hepatomegaly (favoring a protruding abdomen), along with hyperlipidaemia, hyperuricemia, and lactic acidemia, besides, hepatomegaly is worsened by liver fat accumulation (Chen, 2001; Chou et al., 2002; Figure 3B). In GSD-Ib patients, short stature, xanthomas, and diarrhea have also been reported; additionally, fasting hypoglycemia may cause seizure. Signs and symptoms of the disorder generally develop during the childhood, around the age of 3 or 4 months, when babies start to sleep through the night, not eating as frequently as newborns. Affected children have a typical aspect with puffy cheeks and doll-like facies (Bartram et al., 1981). Untreated GSD-Ib is childhood lethal (Chou and Mansfield, 2011). Long-term complications include growth retardation, delayed puberty, osteoporosis, pancreatitis, gout, pulmonary hypertension, polycystic ovaries, and increased risk of hepatocellula adenoma (Chou et al., 2002, 2010b; Rake et al., 2002).

GSD-Ib myeloid phenotype is shared with GSD-Irs. A faulty G6PT/G6Pase-β complex activity causes neutrophil dysfunction and congenital neutropenia, therefore either GSD-Ib or GSD-Irs patients suffered from recurrent infections.

**Figure 4:**
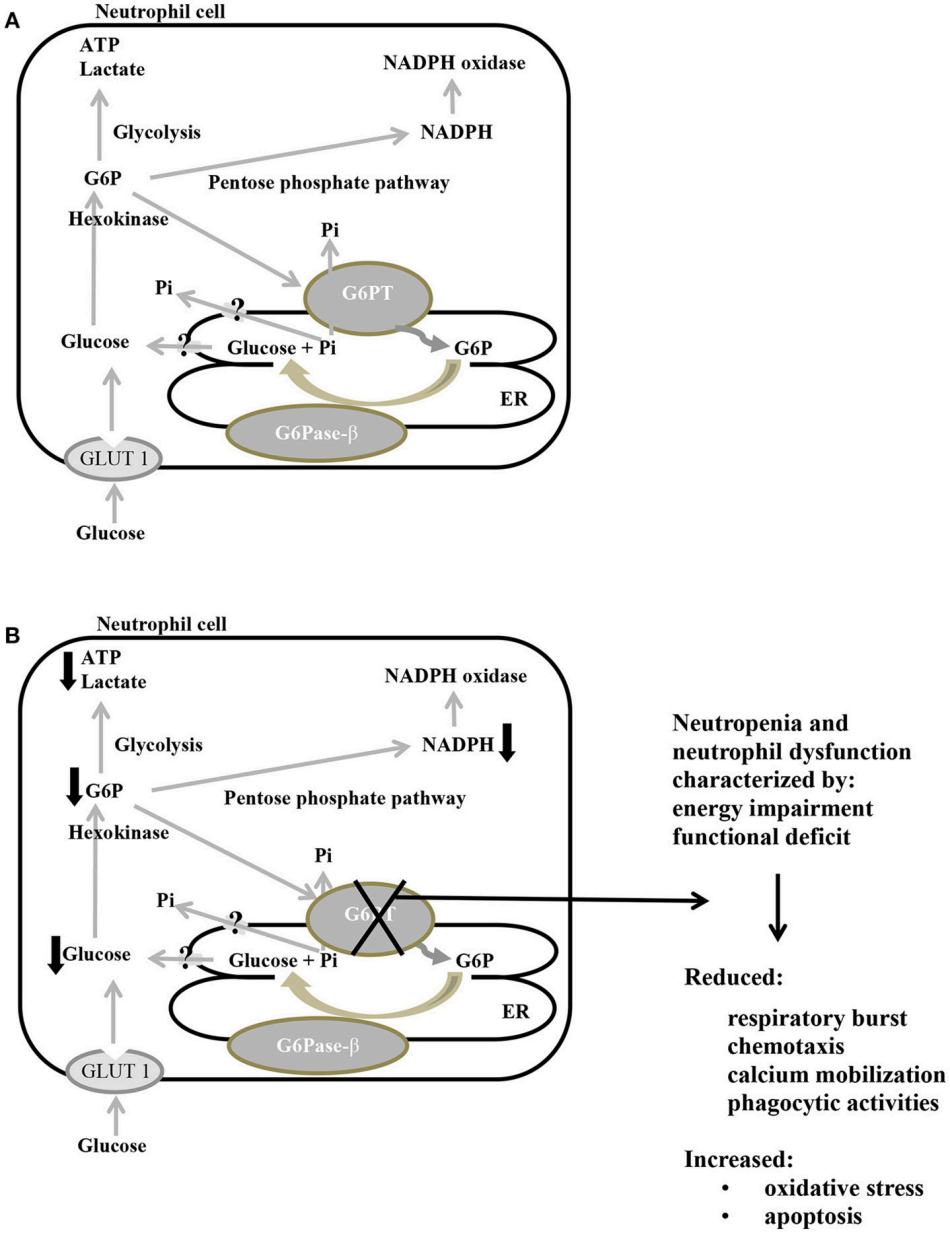
Main metabolic pathways of G6P in normal **(A)** and defective G6PT **(B)** neutrophils. Schematic cell showing an extended endoplasmic reticulum (ER) and the three major pathways (glycolysis, pentose phosphate pathway, and ER cycling) in which G6P is involved. G6Pase-β and G6PT are embedded in the ER membrane; GLUT 1 is embedded in the plasma membrane. Black arrows indicate metabolic changes due to defective SLC37A4.: G6P, glucose-6-phosphate; G6Pase-β, glucose-6-phosphatase-β; G6PT, glucose-6-phosphate translocase; GLUT 1, glucose transporter 1; P, phosphate; Pi, inorganic phosphate; ATP, adenosine triphosphate; NADPH, nicotinamide adenine dinucleotide phosphate.

In neutrophils, glucose imported into the cytoplasm via GLUT1 is metabolized by hexokinase to G6P, which in turn enters the ER lumen through G6PT, where it can accumulate until it is hydrolyzed to glucose by G6Pase-β and transported back into the cytoplasm. Intracytoplasmic G6P/glucose ratio is affected by several pathways, such as glycolysis, pentose phosphate pathway, and recycling of G6P/glucose between the ER lumen and the cytoplasm (Jun et al., 2010; Figure 4A). The G6PT/G6Pase-β complex plays a key role in the third pathway, because glucose recycling decreases cytoplasmic G6P/glucose ratio, so regulating the previously mentioned cytoplasmic pathways for G6P metabolism. As a consequence, G6PT impairment arises a lack of glucose recycling that can cause impaired neutrophil, macrophage, and monocytes functionality, as well as energy homeostasis, leading to reduced intracellular levels of G6P, lactate, ATP and NADPH (McCawley et al., 1993; Jun et al., 2010). A defective G6PT can also cause reduced neutrophil respiratory burst, chemotaxis, calcium mobilization and phagocytic activities (Figure 4B; Kilpatrick et al., 1990; Chou et al., 2010a; Jun et al., 2014).

Furthermore, in G6PT-deficient neutrophils, reduced respiratory burst was associated with an impaired activation of NADPH oxidase, a multicomponent enzyme promoting the production of reactive oxygen species (Jun et al., 2014). Neutrophil metabolism is dependent on anaerobic glycolysis for ATP production. Under hypoxia the protein levels of the hypoxia-inducible transcriptional factor-1α (HIF-1α) increase (Bárdos and Ashcroft, 2005), and neutrophils display defective respiratory burst activity (McGovern et al., 2011). HIF-1α is also an upstream activator of peroxisome proliferator-activated receptor-γ (PPAR-γ) (Krishnan et al., 2009), a nuclear receptor involved in the regulation of lipid and glucose metabolism, influencing inflammation and many other diseases (Kvandova et al., 2016). It was observed that in neutrophils PPAR-γ is constitutively expressed, and its activation leads to chemotaxis inhibition (Reddy et al., 2008). On this basis, it was supposed that the activation of the HIF-1α/PPAR-γ pathway in neutrophils of GSD-Ib patients could trigger neutrophil dysfunction, impairing chemotaxis and calcium mobilization activities (Jun et al., 2014).

GSD-Ib patients may also experience oral symptoms, consisting of dental caries, periodontal diseases, gingivitis, delayed dental maturation and eruption, oral bleeding diathesis and ulcers (Mortellaro et al., 2005). Remarkably, not all GSD-Ib patients manifest neutropenia or frequent infections (Kure et al., 2000; Melis et al., 2005; Angaroni et al., 2006; Martens et al., 2006). In this regard, in a multicentre study investigating the genotype/phenotype correlation on a cohort of 25 GSD-Ib patients, no correlation was found between individual mutations and the presence of neutropenia, bacterial infections or systemic complications. This evidence might suggest the existence of unknown factors able to influence immune phenotype, such as polymorphisms, proteins or genes, capable of modulating neutrophil differentiation, maturation, and apoptosis (Melis et al., 2005). Considering that neutrophils of GSD-Ib patients exhibited enhanced apoptosis, a causal relationship between apoptosis and neutropenia was hypothesized (Kuijpers et al., 2003; Jun et al., 2014). This theory was supported by further studies conducted on animal models, demonstrating that either neutrophils from *G6Pase*-β ^−/−^ mice or those from *G6PT* ^−/−^ mice exhibited enhanced ER stress and apoptosis (Cheung et al., 2007; Kim et al., 2008). So, neutrophil ER stress, higher oxidative stress and apoptosis might be underlying causes of neutropenia in GSD-Ib (Jun et al., 2010). In addition, neutrophil apoptosis in both *G6Pase*-β ^−/−^ (Jun et al., 2011) and *G6PT* ^−/−^ (Kim et al., 2008) mice was mediated by the intrinsic apoptosis pathway. In GSD-Ib, a maturation arrest seems not to be responsible for neutrophil dysfunction (Jun et al., 2014). Since neutrophils, as well as macrophages, have to import glucose from the blood by the glucose transporters (Pessin and Bell, 1992), these cells critically depend on the G6PT/G6Pase-β complex activity, especially when the need for glucose rises (Jun et al., 2014). This might provide a rationale for neutropenia caused by enhanced neutrophil ER stress, oxidative stress and apoptosis, when G6PT and/or G6Pase-β are defective (Kuijpers et al., 2003; Kim et al., 2008; Jun et al., 2010, 2014).

## Autoimmunity risk of SLC37A4 defect

A strong association was found between GSD-Ib and inflammatory bowel disease (IBD), because many GSD-Ib patients with chronic gastrointestinal inflammation were diagnosed with IBD, which was clinically indistinguishable from idiopathic Crohn disease, assuming the involvement of an impaired mucosal innate immunity in the pathogenesis of IBD (Dieckgraefe et al., 2002). The association between neutropenia and IBD was supported by Visser et al., in a retrospective European study, showing that up to 77% of patients with GSD-Ib presented also neutropenia, as well as many GSD-Ib patients suffering from perioral or perianal infections (Visser et al., 2000).

In patients with GSD-Ib an increased risk for Crohn-like disease was reported (Melis et al., 2003), along with a severe deficit of hypothalamus-pituitary-thyroid axis, causing an increased prevalence of thyroid autoimmunity and hypothyroidism (Melis et al., 2007). In one patient, the occurrence of myasthenia gravis was also described (Melis et al., 2008). These observations paved the way to the hypothesis that GSD-Ib patients, but not those affected by GSD-Ia, were at increased risk for autoimmune disorders. The different risk degree between GSD-Ia and GSD-Ib patients might be related to the presence of neutropenia and neutrophil dysfunction only in GSD-Ib (Melis et al., 2007). Some studies have also suggested an association between lymphopenia and autoimmunity (Merayo-Chalico et al., 2016). Chronic lymphopenia might promote autoimmunity inducing the homeostatic expansion of T cell, which represents a normal compensatory reaction during lymphopenic conditions (King et al., 2004).

Recent studies have highlighted the molecular mechanisms underlying the increased frequency of autoimmunity disorders shown in GSD-Ib patients (Melis et al., 2017).

Regulatory T cells (Tregs) are a subset of CD4+ T cells involved in maintaining tolerance to self-antigens, and they play a critical role in human autoimmune diseases, because their imbalance seems to be responsible for the failure of local regulatory mechanisms (Dejaco et al., 2006). Those cells mediate their suppressive function by acting directly on conventional T cells (Tconvs) and antigen-presenting cells, such as dendritic cells (Rueda et al., 2016). Tregs express the transcription factor forkhead box P3 (FOXP3), which is very important for Tregs development and function, since defects in the FOXP3 gene cause a human lethal autoimmune disease (Bennett et al., 2001).

Treg responses can be modulated by several metabolic pathways (Buck et al., 2015), particularly by glycolysis, able to affect human Treg induction and function (De Rosa et al., 2015). In this regard, a defective engagement of glycolysis upon suboptimal T cell receptor (TCR) stimulation of conventional T cells (Tconvs) has been observed in several human autoimmune diseases, and it has been associated with a decreased suppressive function of Tregs (De Rosa et al., 2015).

In GSD-Ib, a defective G6PT causes a decreased capacity to mobilize glucose, leading to diminished glucose utilization. Consequently, a reduced engagement of glycolysis in T cells occurs upon suboptimal TCR stimulation; additionally, qualitative and quantitative alterations of peripheral regulatory T cells (pTregs) have been observed, along with impaired FOXP3 expression by Tconvs during low TCR activation (Melis et al., 2017).

## Therapeutic approaches for SLC37A4 defect

Clinical therapies consist of dietary treatment, granulocyte colony-stimulating factor (G-CSF) therapy and transplantations.

Since recurrent hypoglycemia is responsible for lactic acidosis, hyperuricemia and hypertriglyceridemia, the primary goal of nutritional therapy is to achieve a stable normoglycemia. In addition, dietary treatment aims to reduce metabolic impairment, delaying some of the long-term GSD-Ib consequences. Unfortunately, elevated triglycerides and cholesterol blood levels may persist regardless of diet, along with their consequences (Cappello et al., 2016); moreover, even if nutritional therapy can improve long-term outcomes for many patients, several long-term complications remain. Recommended diet composition is 60–70% calories from carbohydrates, 10–15% from proteins and the remaining from fats (Goldberg and Slonim, 1993). Small frequent feedings rich in complex carbohydrates (particularly those higher in fiber) have to be regularly ingested over 24 h to achieve a good metabolic control (Wolfsdorf and Weinstein, 2003; Kishnani et al., 2014). GSD-Ib, as well as GSD-Ia patients, can use the same nutritional therapy to avoid hypoglycemia, especially nighttime (Chou et al., 2010b).

Cornstarch therapy in GSD-I is used since the early 1980s (Chen et al., 1984). Raw uncooked cornstarch is a slowly digested carbohydrate, which allows a slow release of glucose able to prolong the length of euglycemia between meals (Sidbury et al., 1986).

Glucose requirement normally decreases with age, so adults have a longer fasting tolerance with respect to children. Traditionally, in children older than 3 years, as well as in adults, normoglycemia during nighttime is maintained by the ingestion at bedtime of uncooked corn starch (Wolfsdorf and Crigler, 1997; Weinstein and Wolfsdorf, 2002; Shah and O'Dell, 2013) or of modified forms of corn starch (Bhattacharya et al., 2007; Correia et al., 2008). In children <3 years continuous tube feeding is usually needed (Greene et al., 1976), because the level of pancreatic amylase able to hydrolyze raw starch is low before 3 years of age (Chou et al., 2010b). A recent controlled crossover study enrolling adult GSD-Ia and GSD-Ib patients, highlighted that nighttime glucose control can be achieved also by the ingestion of a cooked pasta meal at bedtime, allowing a more palatable alternative to corn starch use (Hochuli et al., 2015).

G-CSF administration promotes the production of granulocytes (Mehta et al., 2015), increasing neutrophile numbers and reducing the frequency and severity of infections in GSD-Ib patients, but it can not improve neutrophile dysfunction (Visser et al., 2002). G-CSF also appears to increase neutrophil survival (Jun et al., 2011). In this regard, studies led *in vivo*, on murine *G6Pase*-β-deficient models, highlighted that G-CSF therapy was able to normalize neutrophil energy homeostasis and to improve functionality, as supported by improved neutrophil glucose uptake and increased G6P, ATP, and lactate intracellular concentrations (Jun et al., 2011).

A recent study has evidenced a partial improvement of neutrophil respiratory burst activity in a GSD-Ib patient by oral administration of galactose, even though the role of galactose in sugar metabolism of GSD-Ib neutrophils remains to be clarified (Letkemann et al., 2017).

Neutropenia associated with IBD can be treated by using a combination of G-CSF and 5-aminosalicylic acid (Visser et al., 2002; Koeberl et al., 2009; Chou et al., 2010b; Shah and O'Dell, 2013).

In some GSD-Ib patients, metabolic abnormalities can be corrected by liver or combined liver/kidney transplantation, since such operations have improved metabolic control, restoring normal fasting tolerance (Boers et al., 2014). Patients with impending renal failure are suggested to consider combined liver/kidney transplantation (Labrune, 2002). In GSD-Ib patients, myeloid dysfunctions can be treated by bone marrow transplantation, which appears to be a promising approach, even if it requires further validation (Pierre et al., 2008). According to the guidelines from the European study on GSD-I, liver transplantation is suggested in GSD-I patients with unresectable hepatocellular adenomas unresponsive to nutritional therapy, mainly if adenomas are associated with serious compression or hemorrhage, or in the case of enhanced risk of transformation into hepatocellular carcinomas (Rake et al., 2002). Although liver transplantation improves metabolic impairment (Reddy et al., 2009), its usefulness on renal disease and neutropenia or neutrophil dysfunction is yet to be settled.

## Gene therapy for SLC37A4 defect

Gene therapy represents a promising strategy to correct metabolic abnormalities in all GSD-I patients (Chou and Mansfield, 2011), although currently it seems not to compensate for myeloid and renal dysfunction in GSD-Ib patients (Kwon et al., 2017).

Several gene transfer vectors, including recombinant adeno-associated virus (rAAV) vectors, have been developed and tested, either on GSD-Ia or on GSD-Ib animal models (Chou et al., 2015; Kwon et al., 2017).

*G6PT* knockout (*G6PT*^−/−^) mice are excellent *G6PT*-deficient murine models, since they exhibit all the metabolic and myeloid dysfunctions typical of human GSD-Ib (Chen et al., 2003). Some studies led on those mice showed that recombinant adenovirus-mediated *G6PT* gene transfer was able to deliver the transgene to the liver and bone marrow, improving metabolic and myeloid defects; however, this therapy was short-lived, owing to the fast loss of vector-mediated gene expression (Yiu et al., 2007).

Further studies led on such mice showed that a human *G6PT* expressing recombinant AAV serotypes 2/8 vector (rAAV8) directed by a hybrid chicken β-actin (CBA) promoter and a cytomegalovirus (CMV) enhancer, was able to deliver the transgene primarily to the liver, thus normalizing metabolic abnormalities. Nevertheless, two of the five treated mice survived for 51–72 weeks, developed multiple hepatocellular adenomas, and one experienced cancer transformation (Yiu et al., 2009). So, this therapeutic approach, while allowing normoglicemia, could not prevent long term complications of hepatocellular adenoma.

A further work, on *G6Pase*-α-deficient murine models (manifesting all the symptoms of human GSD-Ia), was led in order to compare two different gene transfer methods. In one case, murine *G6Pase*-α gene transfer *in vivo* was mediated by recombinant AAV serotypes 2/1 (rAAV1) or rAAV8 vectors, and expression was directed by hybrid CBA promoter/CMV enhancer. In the other case, a rAAV8 vector expressing human G6Pase-α directed by its promoter/enhancer (GPE) was employed (Yiu et al., 2010).

This work highlighted that the gluconeogenic tissue-specific human GPE was more effective in directing persistent *in vivo* hepatic transgene expression with respect to the CBA promoter/CMV enhancer, because human GPE utilization triggered a lesser humoral immune response, allowing complete normalization of hepatic G6Pase-α deficiency (Yiu et al., 2010). In addition, in murine GSD-Ia models, the use of a *G6Pase*-α-expressing rAAV vector under the GPE control was able to correct metabolic defects with no hepatocellular adenoma development (Lee Y. M. et al., 2012).

On this basis, recently two different liver directed gene therapy approaches were tested on *G6PT*^−/−^ mice, using rAAV-GPE-*G6PT* and rAAV-miGT-*G6PT*, this latter consisted of a human *G6PT* expressing rAAV directed by human minimal *G6PT* promoter/enhancer (miGT) (Hiraiwa and Chou, 2001; Kwon et al., 2017).

Both vectors could deliver the *hG6PT* transgene to the liver, correcting metabolic anomalies, but rAAV-GPE-*G6PT* vector directed by the *hG6Pase*-α promoter/enhancer had greater efficacy. A full restoration of normal G6PT activity was not necessary to obtain significant therapeutic benefits, since a restoration of 3-62% seemed to confer protection against age-related obesity and insulin resistance, while restoration <6% exposed to hepatic cancer risk (Kwon et al., 2017).

## Author contributions

AC, RC and RL conducted literary review, wrote the article and prepared publication-ready figures, MM and VD designed and edited the article.

### Conflict of interest statement

The authors declare that the research was conducted in the absence of any commercial or financial relationships that could be construed as a potential conflict of interest.

## Abbreviations

AMPK: 5′ AMP-activated protein kinase
ATG: autophagy-related gene
CBA: chicken β-actin
CRC: colorectal cancer
WAT: white adipose tissue
CHI: congenital hyperinsulinism of infancy
Tconvs: conventional T cells
FOXP3: forkhead box P3
G6Pase-α: promoter/enhancer GPE
G6Pase-system: glucose-6-phosphatase system
G6PT: glucose-6-phosphate translocase
GSD-I: glycogen storage disease type I
G-CSF: granulocyte colony-stimulating factor
HIF-1α: hypoxia-inducible transcriptional factor-1α
IBD: inflammatory bowel disease
mTORC1: mammalian target of rapamycin complex 1
miGT: minimal G6PT promoter/enhancer
OPA: organophosphate/phosphate antiporter
PPAR-γ: peroxisome proliferator-activated receptor-γ
phospho-Ser294 PR: phospho-Ser294 progesterone receptor
CMV: cytomegalovirus
rAAV: recombinant adeno-associated virus
Tregs: regulatory T cells
RUNX2: runt-related transcription factor 2
SLC: solute-carrier
SP: stabilizing protein
SPX: sugar-phosphate exchangers
TCR: T cell receptor
ULK1: unc-51 like autophagy activating kinase 1
VDR: vitamin D receptor.
